# The Effects of Isoproterenol and Propranolol on Cytokine Profile Secretion by Cultured Tumor-infiltrating Lymphocytes Derived from Colorectal Cancer Patients

**Published:** 2011-12-22

**Authors:** Shahram Seyedi, Alireza Andalib, Abbas Rezaei, Seyed Mohsen Hosseini, Seyed Reza Mohebbi, Mohammad Reza Zali, Mohamad Vafai, Roubik Behboo, Seyed Abbas Tabatabaei, Shahram Shahabi

**Affiliations:** 1. Department of Immunology, Isfahan Medical School, Isfahan University of Medical Sciences, Isfahan, Iran; 2. Department of Epidemiology and Biostatistics, School of Health, Isfahan University of Medical Sciences, Isfahan, Iran; 3. Research Center for Gastroenterology and Liver Diseases, Shahid Beheshti University of Medical Sciences, Tehran, Iran; 4. Iranian Scientific Society of Osteoma Care, Tehran, Iran; 5. Department of Thoracic Surgery, School of Medicine, Isfahan University of Medical Sciences, Isfahan, Iran; 6. Department of Microbiology, Immunology and Genetics, Faculty of Medicine, Urmia University of Medical Sciences, Urmia, Iran

**Keywords:** Isoproterenol, Propranolol, Beta-2 Adrenergic Receptor (β-2AR), Tumor-infiltrating Lymphocytes, Colorectal Cancer

## Abstract

**Objective:**

Anti-tumor immunity and cytokine profiles have important roles in the development of cancer. Norepinephrine (NE) release due to sympathetic activation leads to a Th2 deviation via the beta-2 adrenergic receptor Beta-2 adrenergic receptor (β-2AR) and could increase cancer progression. This study intends to determine the effects of isoproterenol (ISO; beta-agonist) and propranolol (PRO; beta-antagonist) on the production of IFN-γ, IL-4, and IL-17. Cytokine levels have been examined in tumor-infiltrating lymphocytes (TILs) and peripheral blood mononuclear cells (PBMCs) of patients with colorectal cancer (CRC). The β-2AR expression on lymphocyte subsets was also assessed.

**Materials and Methods:**

In this experimental study, TILs were isolated from fresh CRC tissue and patient PBMCs were obtained just prior to surgery. The cells were cultured in medium for 72 hours. Concomitantly, cells were stimulated with 10 µg/ml phytohemagglutinin (PHA) alone or in the presence of either 1 µmol/L of PRO or 1 µmol/L ISO. The concentration of cytokines in the supernatants was measured by ELISA. Three-color flow cytometry was used to determine the expression of β-2AR on the lymphocyte subsets. Statistical analyses were performed via paired or independent t-test.

**Results:**

Levels of IFN-γ, IL-4 and IL-17 were elevated after PHA-stimulation of PBMCs and TILs. However, the elevation of IFN-γ and IL-17 production by TILs in response to PHA was significantly lower than PBMCs. In the presence of ISO, the IFN-γ/IL-4 ratio reduced in all groups, but this reduction was very low in TILs. Interestingly, the effects of PRO on cytokine production were, at least partially, comparable to those of ISO. Depressed levels of β-2AR expression were demonstrated on CD4+IFN-γ+ and CD4+IL-17+ lymphocytes in patients' PBMCs and TILs.

**Conclusion:**

This study has demonstrated the effects of ISO and PRO on cytokine production by TILs and determined β-2AR expression on these cells. ISO failed to induce a shift toward the expected Th2 cytokine profile in CRC patients' TILs, which might be due to the downregulation of β-2AR expression on TILs. Additionally, in this study, PRO induced a shift to a Th2 profile in PBMCs.

## introduction

Colorectal cancer (CRC), one of the most frequent and aggressive cancers, is the fourth leading cause of cancer deaths worldwide (1, 2). Current treatments for CRC include surgical resection (the treatment of choice), as well as radiotherapy and chemotherapy, which are curative in many patients (3). New therapeutic strategies (e.g., immunotherapy), however, are needed to improve survival (4).

Interactions between tumors and the immune system, especially in the tumor microenvironment, are dynamic, complex and bi-directional. T-helper lymphocytes (Th), which can be subdivided into IFN-γ-secreting Th1, IL-4-secreting Th2, and IL- 17-secreting Th17 cells, are very important in antitumor immunity. A shift toward a Th1 response results in tumor rejection, whereas a shift toward a Th2 response prevents tumor rejection (5). The role of Th17 cells in tumor immunity and their promotion or inhibition of tumor progression is unknown (6, 7). Thus, determining the role of inflammatory cells and pro-inflammatory cytokines in the tumor microenvironment of CRC is critical (8).

Two major pathway systems are involved in the neuroimmune interaction: the hypothalamicpituitary- adrenal (HPA) axis and the sympathetic nervous system (SNS) (9). Beta2-adrenergic receptor (β-2AR) regulates immune function using binds to norepinephrine (NE) a neurotransmitter of SNS. β-2AR is expressed differentially on immune cells, including natural killer (NK) cells, T cells, and macrophages (10-12). β-2AR stimulation alters proliferation, cytokine production, and the circulation of innate and adaptive immune cells (13). Stimulation of β-adrenergic receptor inhibits the production of TNF-α and IL-1β, and increases IL-10 levels in whole blood in response to lipopolysaccharides (LPS) and IL-10 production in macrophages (14, 15). Thus, it seems that it has an anti-inflammatory effect. In adaptive immunity, NE, β-agonists (i.e., isoproterenol, ISO), and even cAMP-elevating agents decrease the level of IFN-γ in Th1 cells and increase the level of IL-4 in Th2 cells (13). In addition, exposure of human PBMC to NE or a β-2AR agonist causes a decrease in IFN-γ production but an increase in IL-4 and IL-10. It can therefore be suggested that exposure to NE leads to a shift to a Th2-like cytokine profile (10). β-AR activation by agonists, catecholamines or SNS activation causes immunosuppression, especially in cellular immunity (9, 16).

This study investigates the response of lymphocytes to catecholamines, and evaluates the impact of ISO and PRO on cytokine production of TILs and PBMCs derived from CRC patients.

## Materials and Methods

### Patients and controls

This experimental study consisted of 17 CRC patients treated at Bahman and Mehr Hospitals in Tehran, Iran, between 2009 and 2010. Patients had histologically confirmed colorectal adenocarcinoma. There were 10 male and 7 female patients, whose mean age was 56.29 years (range: 40-81 years). All patients underwent surgery . There was no history of any chemotherapy, radiotherapy, or other therapies that influenced the immune system. Patients had no autoimmune diseases.

The control group consisted of 17 healthy volunteers, of which there were 14 men and 3 women with a mean age of 36.94 years (range: 25-48 years).

The study protocol was approved by the Ethical Committee of Isfahan University of Medical Sciences. An informed consent was signed by all subjects enrolled in the present study.

### Materials

RPMI-1640 medium, PBS tablets , trypan blue, (±) propranolol hydrochloride (PRO) and (−) isoproterenol hydrochloride (ISO) were purchased from Sigma (USA). Penicillin, streptomycin, and fetal bovine serum (FBS) were purchased from Invitrogen (USA). ID ELISA Human IFN-γ, IL-4 and IL-17 ELISA Kit were purchased from Idlabs (Canada).

### Antibodies and reagents

FITC anti-human IFN-γ (clone 4S.B3), FITC antihuman IL-4 (clone MP4-25D2), FITC anti-human IL-17 (clone eBio64DEC17), PE donkey F(ab')2 anti-rabbit IgG (polyclonal, which was used as the secondary antibody) and isotype controls, that included FITC mouse IgG1 (clone P3.6.2.1), FITC rat IgG1, and PE mouse IgG1 (clone P3.6.2.1) were purchased from eBioscience (USA). PerCP anti- CD4 antibody (clone MEM-241), PE anti-CD3 antibody (clone BB12), and β-2AR antibody (polyclonal) were purchased from Abcam (USA). The BD Cytofix/Cytoperm Plus Kit (with BD GolgiPlug) was purchased from BD Biosciences (USA).

### Isolation of TIL

Preparation of TIL was performed by the method described by Rosenberg, with some modifications. Briefly, tissue samples were taken from the tumor mass immediately after surgical removal. Samples were placed in tubes filled with phosphate buffered saline (PBS) at 4℃ and prepared for analysis within 1 hour. Fat tissues were physically removed. The specimens were then cut into small pieces and homogenized with a homogenizer. The resulting cell suspensions were filtered through sterile gauze and then a nylon mesh (100 µm) into centrifuge tubes. After several washes, the cells were placed on lympholyte-H (Cedarlane, Burlington, NC, USA) and centrifuged, without braking, for 20 minutes at 800 g and 20℃. TIL were removed from the interface and resuspended at a concentration of 1×10^5^ cells/ml in complete media, which consistd of RPMI 1640 medium supplemented with 10% FBS, penicillin (100 units/ml), streptomycin (100 µg/ml), and gentamicin (40 µg/ml). Cell viability was determined by trypan blue staining.

### PBMC isolation

Heparinized blood was obtained from healthy controls and patients just before surgery. Mononuclear cells were separated by the Ficoll-Hypaque gradient centrifugation technique. Peripheral blood samples were diluted with an equal volume of PBS. Cell suspensions were layered over lympholyte-H and centrifuged at 800 g for 20 minutes at 20℃ without braking to separate PBMCs. Cells at the gradient interface were collected, washed twice and then resuspended in RPMI-1640. Viability and cell counts were determined.

### Three-color staining and flow cytometry

Healthy controls' PBMCs, patients' PBMCs and patients' TILs were seeded at 1×10^5^ cells into 24-well culture plates (BIOFIL, USA) in 1 ml of RPMI-1640. Cells were stimulated with 10 µg/ml phytohemagglutinin (PHA) for 10 hours and incubated at 37℃ in a humid atmosphere that contained 5% CO_2_. After 4 hours of stimulation, 1 µl of GolgiPlug (from the BD Cytofix/Cytoperm Plus Kit with BD GolgiPlug) was added.

Cells were then centrifuged for 5 minutes at 600 g, resuspended in PBS, and stained for surface antigens and intracellular cytokines. Briefly, cells were incubated with 10% human serum for 15 minutes at 4℃. The cell suspensions were labeled with fluorochrome-conjugated antibodies against cell surface antigens for 30 minutes in the refrigerator in the dark. The β-2AR primary antibody was added at this stage. After washing, cells were labeled with PE donkey F(ab')2 anti-rabbit IgG as the secondary antibody for 30 minutes at 4℃ in the dark. Washed cells were resuspended and 250 µl of Fix/Perm solution added for 20 minutes at 4℃. Cells were washed with 1 ml BD Perm/ Wash buffer and centrifuged. Cells were resuspended in BD Perm/Wash buffer and stained with anti-cytokine antibody for 30 minutes at 4℃ in dark. Finally, cells were washed twice with 1 ml BD Perm/Wash buffer and resuspended in PBS for flow cytometric analysis. Flow cytometry was performed by FACScalibur (Becton Dickinson, Mountain View, CA), and CellQuest Pro software was utilized for data analysis.

A histogram of the fluorescence distribution of β-2AR in different lymphocyte subsets was constructed, and the relative geo mean fluorescence intensity (MFI) was obtained from the histogram and expressed as an index of membrane surface expression. To show lymphocyte subsets, cells were first gated by their physical properties (FSC and SSC), and a second gate was then set based on the fluorescence characteristics of the gated cells. Background staining was assessed using samples incubated with only the PE-conjugated secondary antibody.

### Cell cultures, isoproterenol and propranolol treatment

PBMCs and TILs from each patient, and PBMCs from controls were incubated in RPMI 1640 medium that contained 10% FBS and antibiotics in 24 well plates. Cells, at a concentration of 10^5^ cells/well, were incubated at 37℃ and 5% CO_2_ for 72 hours to evaluate cytokine production. We designed four treatment conditions for the cell populations: i. cells grown in complete culture media without any stimulation, Iso or Pro; ii. cells cultured in complete culture media with 10 µg/ml PHA; iii. cells stimulated with PHA, and 1 µmol/L PRO only added at the time of activation; and iv. cells stimulated with PHA and 1 µmol/L ISO only added at the time of activation. After 72 hours, culture supernatants were collected and stored at -70℃ until the cytokine assay.

### Cytokine assay

IFN-γ, IL-4, and IL-17A levels were determined by sandwich ELISA. All procedures were performed as described by the manufacturer. Briefly, standards and samples were added to 96-well cytokine coated plates. Wells were washed and detection antibody was added. Avidin-HRP and TMB substrate were used to complete the process. The wells were read at a wavelength of 450 nm by a colorimetric plate reader (Biotek Instruments Inc., Winooski, VT).

### Statistical analysis

Results are presented as means ± standard deviations (SD). The paired t-test was used to measure the effect of PHA, ISO or PRO on cytokine production. The general linear model (GLM) multivariate analysis was done to see the relative effect of confounding factors on dependent variable. The analysis showed that sex and age did not have any significant effect on the variable in our model. Thus, to evaluate differences between groups, we used the independent t-test. The association was evaluated by a Pearson correlation coefficient with SPSS 16.0 software.

## Results

### The pattern of IFN-γ, IL-4 and IL-17 cytokine production in unstimulated PBMCs and TILs after 72 hours of culturing

The amount of IFN-γ produced by PBMCs was significantly higher in patients (207 ± 81 pg/ml) compared with healthy controls (138 ± 37 pg/ml; p=0.009).

The mean IFN-γ concentration in TILs was 398 ± 112 pg/ml, which was higher than that of PBMCs (p<0.001).

The levels of IL-4 in cultured PBMCs was 35.3 ± 9.2 pg/ml in the controls, 38.8 ± 16.2 pg/ml in the patient group (p=0.495), and 41.3 ± 14 pg/ml in cultured TILs, which was not significant (Table 1).

The IL-17 concentration was 1948 ± 412 pg/ml in patients' PBMCs, 615 ± 139 pg/ml in healthy controls (p<0.001), and 1795 ± 431 pg/ml? in TILs. The level of IL-17 in patients was higher than controls.

The ratio of IFN-γ/IL-17 was 0.25 ± 0.1 in TILs and 0.1 ± 0.03 in patients' PBMCs (p<0.001). This ratio was 0.23 ± 0.07 in healthy controls' PBMCs. A positive correlation was observed between the IFN-γ and the IL-17 concentration in patients' PBMCs; however it was negative in TILs.

### The level of IFN-γ, IL-4 and IL-17 cytokine production in culture supernatant in PHA-stimulated PBMCs and TILs after 72 hours

**Fig 1:**
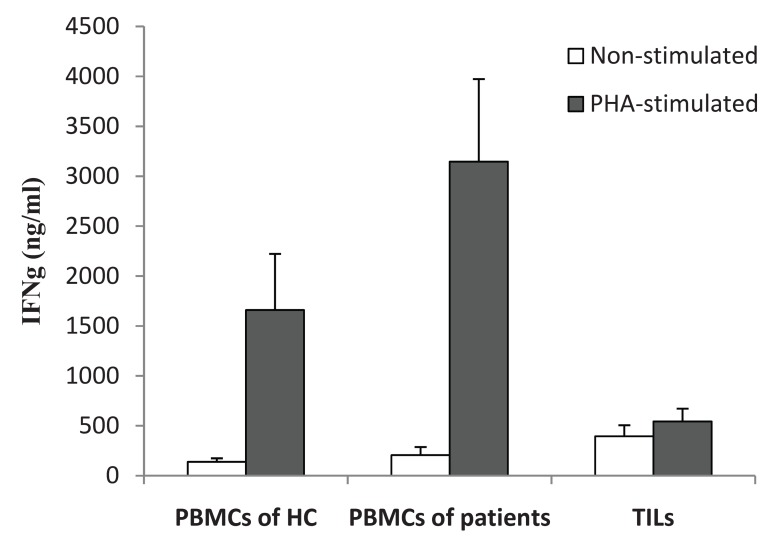
IFN-γ production in response to PHA. HC; healthy control group. TILs; tumor-infiltrating lymphocytes. PBMCs; peripheral blood mononuclear cells.

The levels of all cytokines were elevated to differing degrees in the three groups. Upon PHA stimulation, the level of IFN-γ increased approximately 12- fold in the PBMCs of healthy controls, 20-fold in the PBMCs of patients, and 1.4-fold in TILs (Fig 1).

**Table 1:** Lymphocyte cytokine production in culture supernatant response to PHA


		PBMC of healthy controls (pg/ml) ± SD	PBMC of patients (pg/ml) ± SD	Pvalue between HC & patients	TILs (pg/ml) ± SD	Pvalue between patients & TIL	Pvalue between HC & TIL
IFN-γ	(Cells only)	138 ± 37	207 ± 81	0.009	394 ± 112	0.000	0.000
	(+PHA)	1658 ± 564	3146 ± 827	0.000	543 ± 129	0.000	0.000
	(+PHA+ISO)	168.5 ± 53.8	470.9 ± 85	<0.001	437.2 ± 83.3	0.276	0.000
	(+PHA+PRO)	114.5 ± 20.4	230.9 ± 30.5	<0.001	431.6 ± 111	<0.001	0.000
IL-4	(Cells only)	35.3 ± 9.2	38.8 ± 16.2	0.495	41.3 ± 14	0.675	0.196
	(+PHA)	41.3 ± 17.2	32 ± 7.3	0.089	38.9 ± 8	0.031	0.642
	(+PHA+ISO)	63.8 ± 18.6	62.3 ± 17.2	0.809	78 ± 14.7	0.014	0.035
	(+PHA+PRO)	35 ± 7.8	45 ± 12	<0.05	57.8 ± 10.7	0.006	0.000
IL-17	(Cells only)	615 ± 139	1948 ± 412	0.000	1739 ± 431	0.178	0.000
	(+PHA)	1652 ± 274	4572 ± 1319	0.000	2081 ± 553	0.000	0.013
	(+PHA+ISO)	676 ± 139	2378 ± 624	<0.001	799 ± 100	<0.001	0.010
	(+PHA+PRO)	1126 ± 137	5181 ± 1395	<0.001	1765 ± 130	<0.001	0.000


Therefore, it seemed that the responsiveness of TILs to PHA was much lower than that of PBMCs from either patients or healthy controls.

The levels of IL-4 production, in contrast, did not change upon PHA stimulation in all groups. However, the IFN-γ/IL-4 ratio was significantly lower for TILs after PHA stimulation than for patients' PBMCs after PHA stimulation (14.8 ± 6.3 vs. 105 ± 37, p<0.001; Table 2).

The IL-17 concentration increased 3-fold in PHA-stimulated PBMCs in both healthy and patient samples, but only 1.5-fold in TILs.

### Effects of isoproterenol (ISO) treatment on cytokine production by PBMCs and TILs

**Fig 2:**
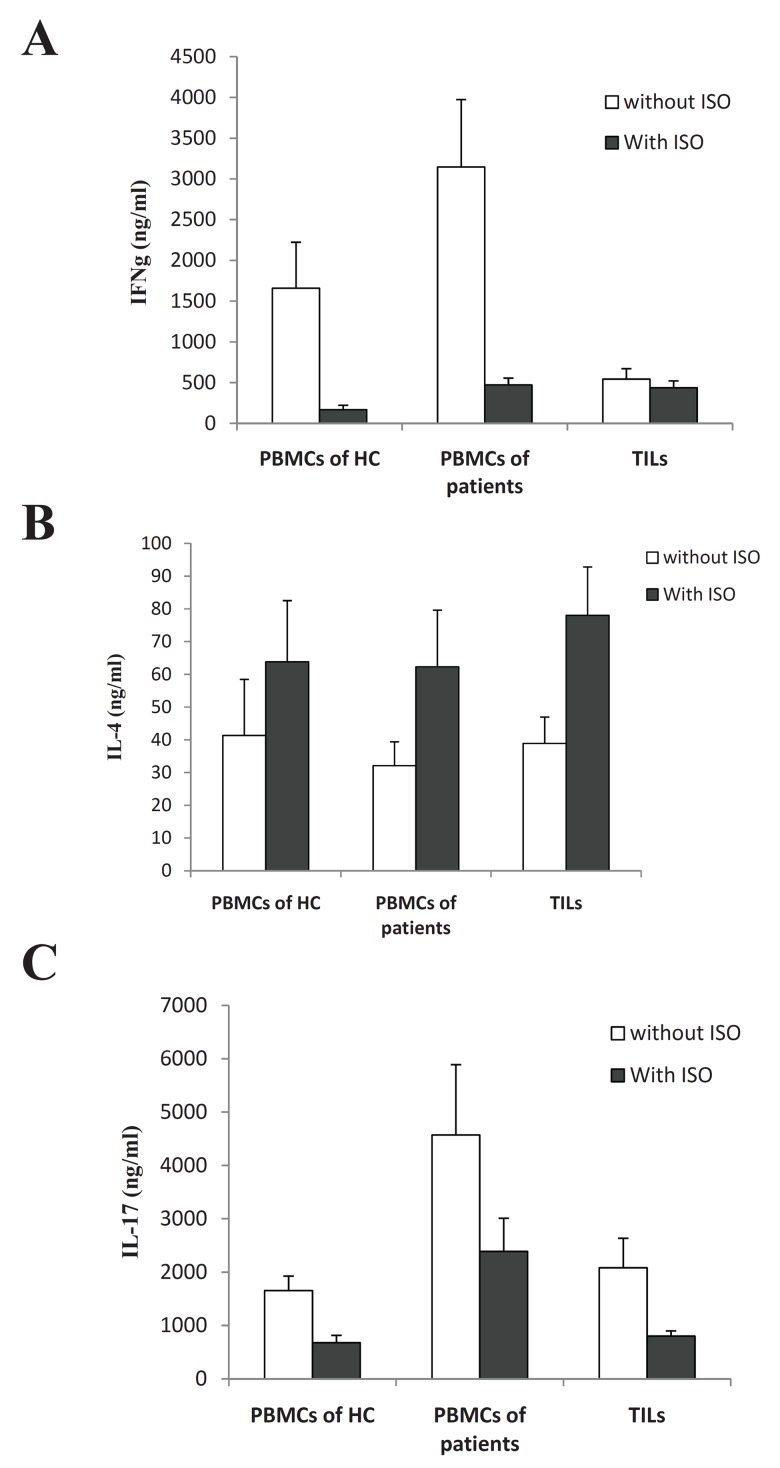
A. The effects of Iso on IFN-γ production, B. The effects of Iso on IL-4 production, C. The effects of Iso on IL-17 production. HC; healthy control group. TILs; tumorinfiltrating lymphocytes. PBMCs;peripheral blood mononuclear cells. ISO;isoproterenol.

As shown in Table 1, ISO in culture medium reduced IFN-γ in all groups (Fig 2A). ISO increased the level of IL-4 in all groups (Fig 2B). In addition, treatment of the cells with ISO led to a decrease in the IL- 17 production in all groups (Fig 2C). However, the IFN-γ/IL-17 ratio in the presence of ISO was reduced in PBMCs and increased in TILs (Table 2).

**Table 2:** Cytokine production ratio (IFN-γ/ IL-4 and IFN-γ /IL-17) in the presence and absence of Isoproterenol(ISO) or propranolol (PRO)


			
IFN-γ/ IL-4			
Without ISO	45.7 ± 26	105 ± 37	14.8 ± 6.5
With ISO	2.8 ± 1.1	7.6 ± 2.3	5.5 ± 1.3
Without PRO	43.7 ± 26.3	105.7 ± 37	14.8 ± 6.5
With PRO	3.4 ± 0.9	5.3 ± 1.4	7.7 ± 2.4
IFN-γ/ IL-17			
Without ISO	1.06 ± 0.33	0.78 ± 0.45	0.27 ± 0.07
With ISO	0.25 ± 0.09	0.22 ± 0.08	0.55 ± 0.09
Without PRO	0.95 ± 0.3	0.57 ± 0.15	0.26 ± 0.07
With PRO	0.1 ± 0.02	0.04 ± 0.01	0.24 ± 0.07


**Table 3:** Mean ± SD for MFI values of β-2AR on lymphocyte subsets in groups


Lymphocyte subsets	PBMC of HC	PBMC of patients	TILs	Pvalue between HC & patient	Pvalue between patient & TIL	Pv between HC & TIL
CD4+IFN-γ+	135.4 ± 40.5	82.7 ± 44	56.9 ± 21	0.001	0.043	0.000
CD4+IL-4+	24.5 ± 4.3	22 ± 6.1	13.7 ± 2	0.170	0.000	0.000
CD4+IL-17+	239 ± 105	105.6 ± 93.9	124.7 ± 66.2	0.000	0.528	0.001


### Effects of propranolol (PRO) treatment on cytokine production by PBMCs and TILs

**Fig 3:**
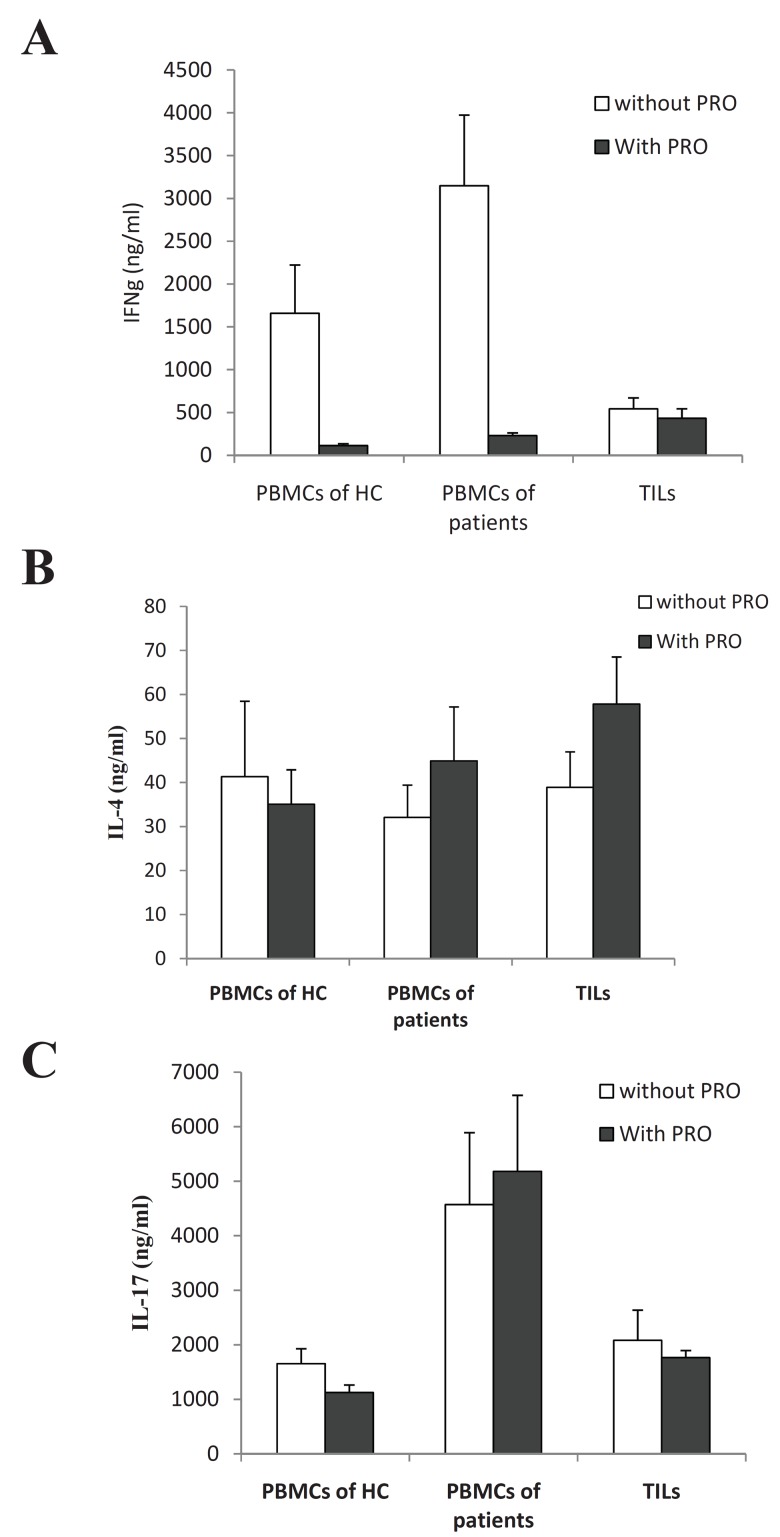
A. The effects of PRO on IFN-γ production, B. The effects of PRO on IL-4 production, C. The effects of PRO on IL-17 production. HC; healthy control group. TILs; tumorinfiltrating lymphocytes. PBMCs;peripheral blood mononuclear cells. PRO; propranolol.

In the presence of PRO there was a significant reduction in the level of IFN-γ in all groups. The reduction was approximately 13-fold in both groups of PBMCs but 1.35-fold for TILs (Fig 3A).

The level of IL-4 slightly increased in the presence of PRO in patients' PBMCs and TILs (Fig. 3B). In the presence of PRO, the IFN-γ/IL-4 ratio reduced in all groups, but the reduction in TILs was considerably lower than in PBMCs of patients and controls (Table 2).

In the presence of PRO, the level of IL-17 decreased by 45% in the PBMCs of healthy controls (p<0.001) and decreased 20% in TILs (p<0.001), but there was no alteration in cancer patients' PBMCs (Fig 3C).

In addition, the IFN-γ/IL-17 ratio was reduced in PBMCs but did not differ in TILs (Table 2).

### Beta-2 adrenergic receptor (β-2AR) expression in lymphocyte subsets

Flow cytometry was performed to evaluate the surface expression of β-2AR in the cell groups. Lymphocytes were gated according to forward and side scatter, and the cell subsets were determined using CD4 (PerCp) vs. cytokine (PE) dot plots. Th1 refers to CD4 + IFN-γ + lymphocytes, Th2 refers to CD4+IL-4+ lymphocytes, and Th17 refers to CD4+IL-17+ lymphocytes. The MFI was used to evaluate surface expression of β-2AR (Table 3). The average MFI values of β-2AR expression on Th1 cells were 135.5 ± 40.5 in healthy controls, 82.7 ± 44 in cancer patients' PBMCs and 57 ± 21 in TILs. The differences between the groups were statistically significant.

The average MFI values of β-2AR expression on Th2 cells did not differ for the PBMCs of healthy controls and cancer patients, but β-2AR expression was lower in the TILs.

The average MFI values of β-2AR on Th17 cells were significantly higher in PBMCs of controls than in patient PBMCs and TILs.

### The relationship between β-2AR expression and cytokine production

The β-2AR expression on Th1 cells correlated negatively with IFN-γ production by non-stimulated PBMCs and TILs (r=-0.509, p<0.01). A similar association was observed for IL-17 and β-2AR expression on Th17 cells (r=-0.518, p<0.001), but not for IL-4 and β-2AR expression on Th2 cells (p=0.351).

## Discussion

One of the most important functional parameters of an anti-tumor immune response is the local production of cytokines. This study evaluated IFN-γ, IL-4 and IL-17, the characteristic cytokines of Th1, Th2 and Th17 cells, respectively.

The level of IFN-γ in TILs was almost 2-fold higher than patients' PBMCs and 3-fold higher than controls' PBMCs. However, the level of IL-4 was not statistically different between groups. These results were consistent with a previous study in which IFN-γ gene expression was 94.1% in colorectal tumor tissue, 84.2% in patients' PBMCs and 40% in controls' PBMCs, with no significant differences noted in IL-4 gene expression (2). A recent study showed that the levels of IFN-γ mRNA were higher in colorectal tumor specimens than normal tissues, but not for IL-4 mRNA (17).

It has been reported that the majority of TILs in CRC are Th1 and T cytotoxic type 1 (Tc1) cells (18). In contrast, there are reports that indicate a reduction in production of IFN-γ or high levels of secreted IL-4 in CRC patients (19, 20). According to research, it has been found that the Th1 immune response is protective in CRC patients (21). It seems this variation among studies could be a reflection of the heterogeneity of tumor development and/or patients' survival, even when the studies involved tumors of the same type

Our results showed that PHA-stimulated cells produced elevated levels of all cytokines when compared to unstimulated cells, but the increase in cytokine production differed between the groups. TILs have a very low response to PHA in terms of cytokine production. The increase of the IFN-γ/ IL-4 ratio in TILs after stimulation with PHA was also lower compared to those of the other groups. This indicateda decreased shift toward a Th1 profile in TILs after PHA stimulation in comparison to the shift of patients' and controls' PBMCs. Defects in cytokine production by mitogen-stimulated lymphocytes have been reported in many conditions (22). This finding is in line with the reported decrease in PHA-induced lymphocyte proliferation in CRC (23). Because the T cell response to PHA is a complex process involving both T cells and accessory cells, we suggest that all cell and soluble costimulation, T cell receptors and signal transduction pathways should be examined in future studies.

In the presence of ISO, the levels of IFN-γ and IL-17 decreased in all cell samples, but with different reduction rates. The reduction rate was significantly lower in TILs compared to PBMCs. Our results showed that ISO increased IL-4 levels and reduced the IFN-γ/IL-4 ratio, which lead to a Th2 deviation in all tested cell samples. These findings have been supported by previous observations that ISO decreased IFN-γ production and increased IL-4 in PBMCs, and that β2-agonists lead to Th2 differentiation of CD4+T cells (12, 24). It has previously been shown that naÏve T cells responded to ISO, whereas effector cells were not responsive (25, 26), and the pattern of TILs responsiveness to ISO in our study was somewhat similar to that of effector cells. This was consistent with several studies that have reported a higher proportion of activated cells in TILs (3, 27). It was noteworthy that, in this study, Th2 deviation due to ISO was considerably slight for TILs.

We also found that ISO reduced IL-17 production in all tested groups, but at different degrees, with the largest effect on TILs. To our knowledge, our findings regarding the effects of ISO on IL-17 production in human lymphocytes was the first of its kind, but studies in mice have yielded differing results. It has been reported that epinephrine can increase serum IL-23 and IL-17 in mice (28) and murine DC exposed to epinephrine can induce IL-17A production by CD4+ T cells (11). This discrepancy could be explained by differences in the development of Th17 cells in humans and mice. Th-17 differentiation is induced by IL-1β and IL-6 in humans, and by TGF- β and IL-6 in mice (29). ISO reduces IL-1β and increases TGF-β in many cells (15, 30). Thus, it is acceptable that ISO decreases IL-17 in humans, but increases IL-17 in mice. Altogether, with regard to lymphocytes and cytokine production, it seems that ISO has a different effect in the tumor microenvironment.

In the presence of PRO, IFN-γ and IL-17 levels reduced and IL-4 increased in all groups (with two exceptions). Our results also showed that PRO has caused a reduction in the IFN-γ/IL-4 ratio in CRC patients. In this regard, PRO seems to act as a Th2-shifting agent in cancer. As the Th1 immune response plays an important role in anti-tumor immunity, we speculate that PRO reduces anti-tumor immunity. This has recently been supported by an epidemiological study by Friedman (31). In addition, it was reported that PRO reduces cytokine secretion by the hypertrophic scar cells (32). In contrast, Fitzgerald has suggested that PRO, as an NE receptor blocker, may suppress cancer cell development (33). This discrepancy might be due to different aspects of the studies. Some focus on the immunological view and cells, whereas others pursue cancer cell development. Of course, it is clearly known that the anti-tumor effects of PRO are not due to beta-AR antagonism. PRO can increase α1-adrenoceptor activity and act as a partial agonist for adrenoceptor (34, 35), which might be responsible for the abovementioned effects of PRO on cytokine production.

An important finding in our study is the downregulation of β-2AR in cancer patients' lymphocytes. We have demonstrated decreased levels of β-2AR expression on CD4+IFN-γ+ and CD4+IL-17+ lymphocytes in patients' PBMCs and TILs. In keeping with our findings, a reduction of β-2AR expression or sensitivity on PBMCs has been observed in rheumatoid arthritis (RA) patients, patients with septic shock, and those exposed to stressful conditions such as caregivers (36, 37). It has been shown that stimulation of β-2AR shifts immune responses toward a Th2 paradigm (10, 16). Therefore, downregulation of β-2AR in cancer patients demonstrates a failure to induce a shift toward a Th2 cytokine profile. The ability to generate a Th1 type response is critical for the optimal function of anticancer lymphocytes (5). Taken together, it seems that depressed β-2AR levels in immune cells is a way to promote anti-tumor immune response. Interestingly, our results have shown that the shifting of TILs toward Th2 due to ISO is lower than that of patients and controls' PBMCs. The downregulation of β-2AR expression on TILs might be considered as one of the mechanisms.

One of the limitations in the current study are the relatively small case numbers in each group. Therefore, we believe that the future studies with larger case number are needed to confirm the findings of the current study.

## Conclusion

This study demonstrated the effects of ISO and PRO on cytokine production by TILs and determined the β-2AR expression on these cells. These findings showed a failure of ISO to induce a shift toward a Th2 cytokine profile in CRC patients' TILs. This failure might have been due to downregulation of β-2AR expression on TILs. We also demonstrated a Th2 shift in CRC patients' immune cells upon PRO treatment. The results further indicated that ISO reduced IL-17 production by human PBMCs.
